# A Rapid Discrimination of Authentic and Unauthentic Radix Angelicae Sinensis Growth Regions by Electronic Nose Coupled with Multivariate Statistical Analyses

**DOI:** 10.3390/s141120134

**Published:** 2014-10-27

**Authors:** Jie Liu, Weixin Wang, Yaojun Yang, Yuning Yan, Wenyi Wang, Haozhong Wu, Zihe Ren

**Affiliations:** 1 School of Chinese Materia Medica, Beijing University of Chinese Medicine, No.6 Wangjing Zhonghuan South Road, Beijing 100102, China; E-Mails: ljtcm@sina.cn (J.L.); yanyuningtcm@163.com (Y.Y.); wangwenyitcm@163.com (W.W.); wuhaozhongtcm@163.com (H.W.); 2 Institute of Forensic Science, Ministry of Public Security, No.17 Muxidi South Street Xicheng District, Beijing 100038, China; E-Mail: wangweixintcm@163.com; 3 Yinkou Institute For Drug, NO.29 Qinghua West Street, Xishi District, Yingkou 115100, China; E-Mail: renzihetcm@163.com

**Keywords:** Radix Angelicae Sinensis, electronic nose, authentic region, multivariate statistical analyses

## Abstract

Radix Angelicae Sinensis, known as *Danggui* in China, is an effective and wide applied material in Traditional Chinese Medicine (TCM) and it is used in more than 80 composite formulae. *Danggui* from Minxian County, Gansu Province is the best in quality. To rapidly and nondestructively discriminate *Danggui* from the authentic region of origin from that from an unauthentic region, an electronic nose coupled with multivariate statistical analyses was developed. Two different feature extraction methods were used to ensure the authentic region and unauthentic region of *Danggui* origin could be discriminated. One feature extraction method is to capture the average value of the maximum response of the electronic nose sensors (feature extraction method 1). The other one is to combine the maximum response of the sensors with their inter-ratios (feature extraction method 2). Multivariate statistical analyses, including principal component analysis (PCA), soft independent modeling of class analogy (SIMCA), and hierarchical clustering analysis (HCA) were employed. Nineteen samples were analyzed by PCA, SIMCA and HCA. Then the remaining samples (GZM1, SH) were projected onto the SIMCA model to validate the models. The results indicated that, in the use of feature extraction method 2, *Danggui* from Yunnan Province and *Danggui* from Gansu Province could be successfully discriminated using the electronic nose coupled with PCA, SIMCA and HCA, which suggested that the electronic-nose system could be used as a simple and rapid technique for the discrimination of *Danggui* between authentic and unauthentic region of origin.

## 1. Introduction

*Danggui* is one of the most popular traditional Chinese medicines, which has been used in Traditional Chinese Medicine (TCM) for thousands of years, mainly to nourish blood, regulate menstruation, promote blood circulation, and relieve pain [1]. It was first cited in the Shennong'herbal classical (200–300 A.D, Han Dynasty), a classical masterpiece of TCM. The official drug of *Danggui* is the roots of Angelica sinensis (Oliv.) Diels (*Angelica ploy morpha maxim.var.sinensis Oliv.*), Umbelliferae. The quality of this kind of *Danggui* has been proven important in clinical applications over thousands of years. Among them, *Danggui* cultivated in Minxian County, Gansu Province, China, is regarded as the authentic herb according to traditional experience and Gansu Province is therefore considered the authentic region (AR) of origin of *Danggui* [2]. However *Danggui* is also cultivated in several other places, such as Yunnan Province and Shandong Province, which are called unauthentic regions (URs) of origin. *Danggui* from UR have been used in clinical trials, and was not effective enough compared with *Danggui* from the AR. Traditionally, AR and UR *Danggui* can be distinguished by experienced *Danggui* farmers, but this method is obviously dependent on highly subjective judgments. Analytical methods to discriminate AR from UR of *Danggui* include thin-layer chromatography (TLC), GC-MS [3], HPLC [4,5], CE-DAD [6], and although these methods have made significant contributions to the studies of *Danggui*, however, they can't effectively distinguish AR *Danggui* from UR material. Therefore, a rapidly applicable and nondestructive analysis method is still needed to discriminate AR *Danggui* from UR *Danggui.*

An electronic nose is used for detecting volatile compounds and it consists of four parts: a sampling system, an array of gas sensors, and a computer with an appropriate pattern-classification algorithm, capable of qualitative or quantitative analysis of complex gases or odors. Sensors, a key part of electronic nose, include quartz crystal microbalances, polymer composites, surface acoustic waves, conductive polymers and calorimetric sensors [7,8].

The measurement principle of an electronic nose is based on the change in electric resistance of the sensors when volatile compounds are present. The metal oxide sensors are semiconductors and are gas-sensitive oxygen in the air is chemisorbed on vacancies in the lattice of the bulk material and removes electrons from the conducting band:

$${\text{sensor}\;\text{electron}\;\text{t}+(1/2)\text{O}}_{\text{2}}\to {\text{O}}^{-}(s)$$

In the presence of a gas or a fragrant molecule (G), this chemisorbed oxygen (O^−^) reacts irreversibly to produce combined molecules (GO):

$$\text{G}(\text{g})+{\text{O}}^{-}(\text{s})\to \text{GO}(\text{g})+\text{sensor}\;\text{electron}$$

The liberated electrons reduce the potential barrier of the oxide grains, which increases the electron mobility. The resistance of the sensors thus decreases in the presence of volatile compounds. The size of the response depends on the nature of the detected molecules, their concentration and the type of metal oxide used. The response time depends on the reaction kinetics, the volume of headspace measured and the flow rate of the gas [9,10].

## 2. Materials and Methods

### 2.1. Experimental Materials

Twenty one samples were collected from their original growing locations. Samples 1–3 from Yunnan Province were collected by Shude Yang, Yunnan University of Traditional Chinese Medicine. Samples 4–20 from Gansu Province were collected by Fude Yang, Gansu University of Traditional Chinese Medicine. Sample 21 from Shandong Province was collected by Weixin Wang. All the samples were verified by Yuning Yan, Beijing University of Traditional Chinese Medicine, Shude Yang and Fude Yang. Details of the samples are listed in Table 1.

### 2.2. Electronic Nose (EN)

A FOX-3000 (Alpha MOS, Toulouse, France) was used in this study. It consists of a sampling apparatus, an array of sensors, an HS-100 autosampler, air generator equipment and software (Alpha Soft V11, Burlington, MA, USA) for data recording and analyzing the data. The sensor array was composed of 12 metal oxide sensors divided into three chambers: T, P and LY [11]. Table 2 shows a list of sensors used and their main applications.

### 2.3. Experiment Procedure

#### 2.3.1. Optimization of Headspace Time and Headspace Temperature

To select the highest intensity response to optimize the main parameters, different headspace times (600, 900 and 1200 s) and headspace temperatures (40, 60 and 80 °C) were investigated. The response intensity corresponding to different headspace times and temperatures are listed in Tables 3 and 4.

Sixty three samples (three samples for each sampling point) were involved in the experiment. Samples (0.1 g) were accurately weighed and placed in a 10 mL glass jar, sealed and loaded in the autosampler tray. The headspace time and temperature were 900 s and 40 °C, respectively. The injection volume was 500 μL, the injection rate 500 μL/s and the stirring rate 250 rpm. The acquisition time was 200 s. When the measurement was finished, the cleaning phase was activated, which lasted 1200 s. The main purpose was to clean the test chamber and return the sensors to their baseline values.

#### 2.3.2. Electronic Nose Response to Samples

**Figure 1.**
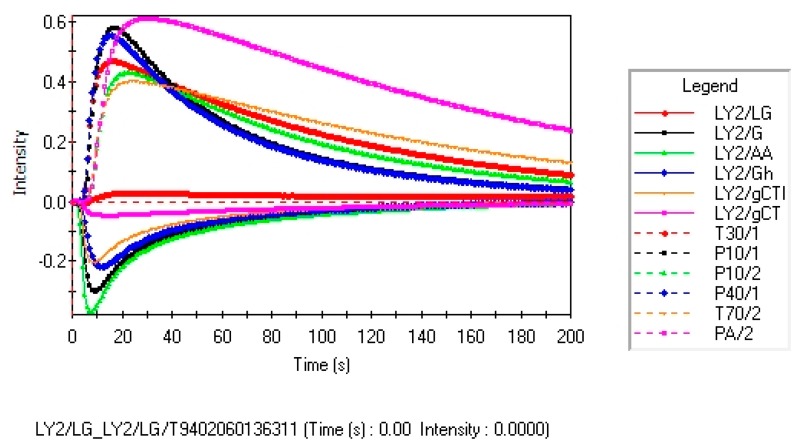
A typical response of 12 gas sensors during the measurement of a sample (GZM1).

Figure 1 shows the typical signal of 12 sensors to sample GZM1. Each curve represents one sensor's conductivity induced by electrovalve action, when a volatile gas reaches the measurement chamber. The EN sensor response of samples, also called odor intensity, is calculated using the following expression [11]:

$$\text{R}=({\text{R}}_{0}-{\text{R}}_{\text{T}}){\text{R}}_{0}$$

where R is the EN sensor response, R_T_ is the value of the conductance of metal oxide sensors, and R_0_ is the value of metal oxide sensors at time 0 s. After a low level in THE initial period, the conductivity increased continuously, and then stabilized after a few seconds.

#### 2.3.3. Repeatability

The repeatability of the sample GZM1 was measured and analyzed in five parallel tests, and the relative standard deviation (RSD, *n* = 5) for each sensor was calculated; the result of each sensor was less than 5%. Details of the repeatability of the GZM1 measurements is listed in Table 5.

#### 2.3.4. Statistical Processing

In this study multivariate statistics methods such as PCA, SIMCA and HCA were used. PCA was applied to determine whether the metal oxide sensor array is able to extract sufficient important information from the table for monitoring the test material [12]. PCA can analyze, classify, and reduce the dimensionality of numerical datasets in a multivariate analysis [13,14]. SIMCA was applied to identify whether each sample belongs to the class or not, according to the established model. SIMCA is a statistical method for supervised classification of data, which provides good or bad, qualified or unqualified results [15]. The method requires a training data set consisting of samples with a set of attributes and their class memberships. Training samples are used to build a model, and they are in the acceptable region and other samples are located outside the acceptable region [16]. A certain sample is projected onto the model to validate the model. If the sample is in the acceptable region, it belongs to the class. If not, the sample is unknown [17,18]. HCA is a standard unsupervised statistical procedure. It provides a better alternative for accurate representation and classification of highly-dimensional data, and it uses the full dimensionality of the data to create a classification dendrogram [11]. HCA was used to study the connections among factors and the scale of each factor.

## 3. Results and Discussion

### 3.1. Feature Extraction Method 1 (FEM1)

The maximum responses of sensors were extracted and analyzed in FEM1. The maximum response was captured because in the combination of sensors in the raw data array, each sensor response curve extremum is similar to the steady state response, related to the amount and properties of gas samples, which is relatively stable and has good repeatability for a set of data. So we chose the average value of the maximum response as the extraction method 1.

#### 3.1.1. Raw Data Analysis

According to the producing provinces, samples were divided into two groups, the Gansu group and Yunnan group. The average value of maximum response of the electronic nose sensors and RSD of each group were calculated. The results are shown in Table 6. The maximum of the absolute value of RSD of *Danggui* in the Gansu group was 23.583%, and the minimum was 9.494%. The maximum RSD of *Danggui* in the Yunnan group was 14.118%, and the minimum was 6.351% (response values below 0.2 were not included). This indicated that the group differences of *Danggui* in the Gansu group were bigger than in the Yunnan group with FEM1. The samples of *Danggui* in the Gansu group were collected from different regions (although all the regions were in Gansu Province), had different storage times and different morphological characteristics and all of these factors may affect the formation of odor characteristics.

#### 3.1.2. Discrimination of Samples Using PCA

**Figure 2.**
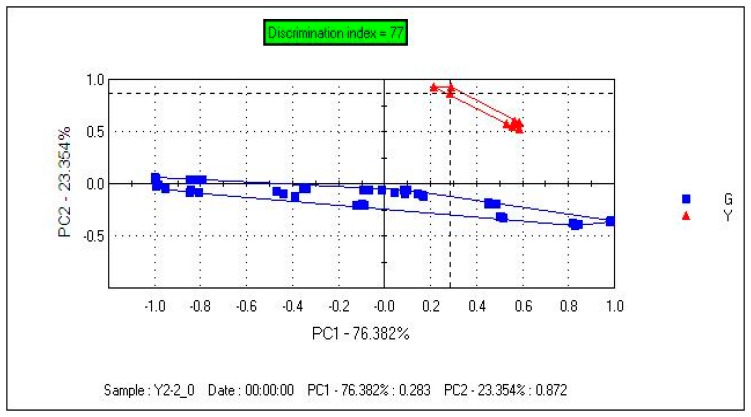
Principal component analysis (PCA) scatter plot for samples (FEM1).

By employing PCA, the total variance does not change with the mathematical transformation. The first variable which has the biggest variance is known as the first principal component (PC1). The second variable, irrelevant to the first variable, called the second principal (PC2). Figure 2 depicts the PCA plot of samples, and the contribution rates of PC1 and PC2 were 76.382% and 23.354%, respectively. The total contribution rate of PC1 and PC2 was 99.736%. From PC1, we can see that *Danggui* in the Gansu group has a large span (length), which indicates that samples in Gansu group have a highly discrete distribution with FEM1 and the differences of samples in the Gansu group is very apparent. From PC1, we can see that the samples in the Yunnan group fell in the Gansu group, we cannot discriminate them from each other; however, by combining PC2, Yunnan group and Gansu group could be clearly separated.

#### 3.1.3. Discrimination of Samples Using SIMCA

**Figure 3.**
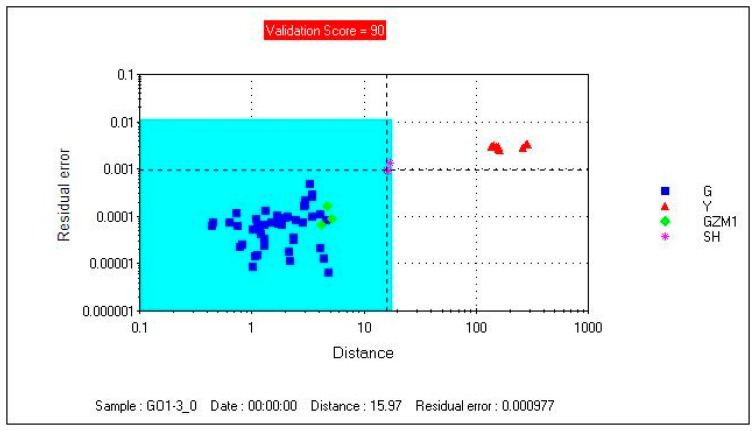
Sample GZM1 and sample SH projected onto the SIMCA model (FEM1).

Figure 3 shows that the model's checking score was 98. The model separated the samples into two regions. The Gansu group was in the acceptable region, and the Yunnan group was outside the acceptable region. GZM1 and SH selected as the unknown samples were projected onto the model which showed that sample GZM1 was correctly project onto the acceptable region and sample SH was incorrectly projected onto the acceptable region. The results indicated that the model was not accurate enough for unknown samples. A more valid feature extraction method should be created.

#### 3.1.4. Discrimination of Samples Using HCA

**Figure 4.**
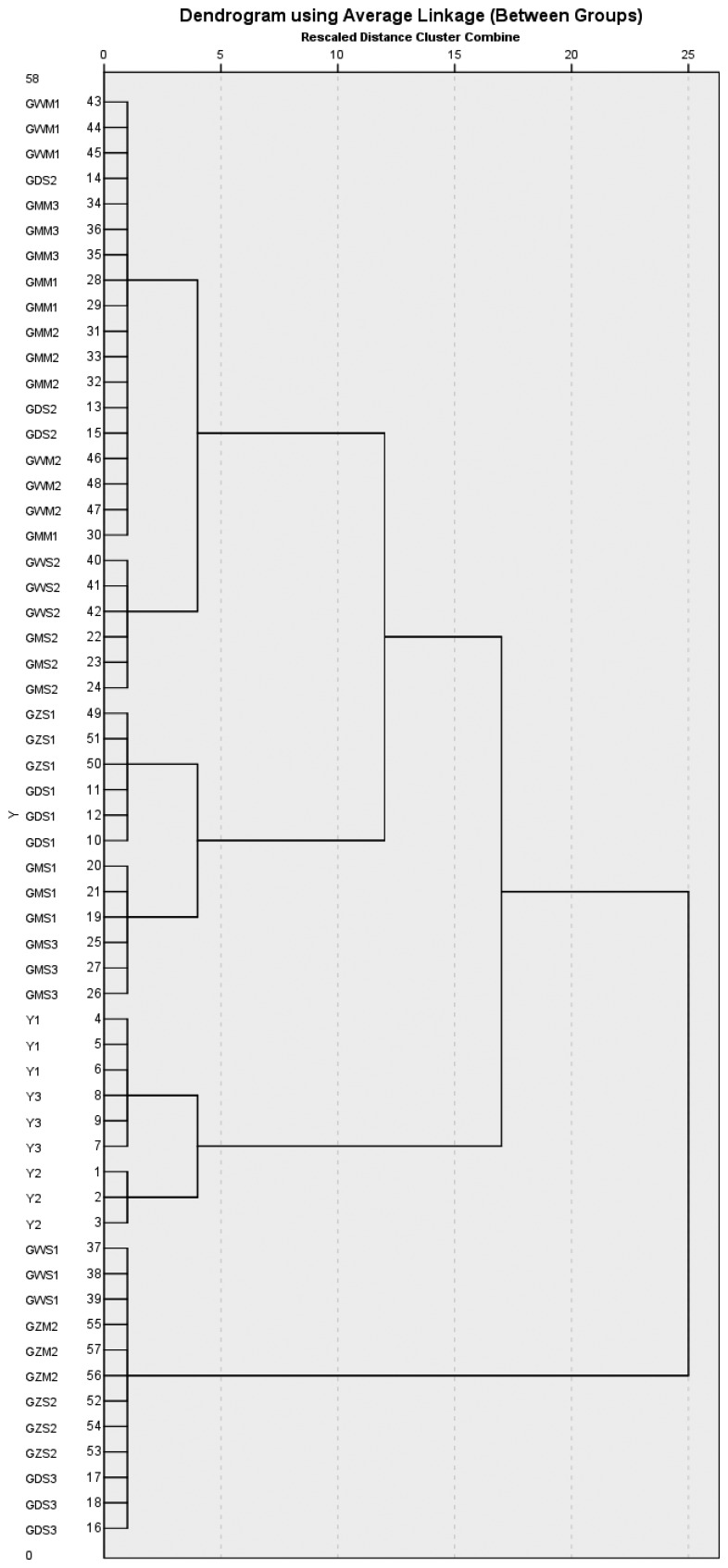
Hierarchical clustering analysis (HCA) dendrogram for samples (FEM1).

The aim of HCA is to divide samples into a specific group by similarity criteria. Figure 4 depicts the dendrogram of HCA, which shows that the samples could be divided into two main different groups, from 3 to 43 was one group, and from 37 to 13 was the other group, however, Y1, Y2 and Y3, which came from Yunnan group couldn't be distinguished from the Gansu group.

### 3.2. Feature Extraction Method 2 (FEM2)

**Figure 5.**
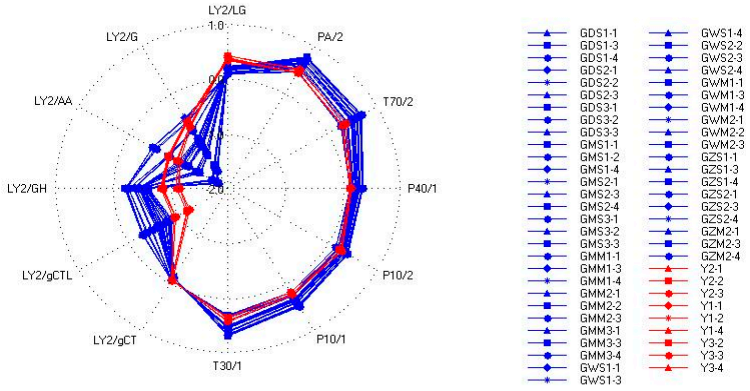
Radar plots for samples.

From above we can see that we can't distinguish Gansu group from Yunnan group successfully with FEM1, so a new feature extraction method should be created. To some extent, the maximum response of sensors may have some limitations in reflecting the characteristics of the TCM odors. Sensors are made up of different precious metals, platinum, palladium, rhodium, and SiO_2_ (coated by precious metals). The differences of metal materials and production processes may affect the response intensity of complex chemical components. The components and their absolute contents together determine the response intensity of each sensor. Due to the particularity of TCM, the same TCM may be seriously affected by many factors, which may make the differences in the absolute content of all kinds of aroma components big, then the response intensities of the sensors also have bigger differences. On the other hand, different odor characteristics are the reflection of aroma components which is the different combination of the amount and types of odors. The ratios between the odor components are important for the combination. Different metal oxide film sensors can sense different olfactory sensitivity components. The response intensity reflects the absolute content of the corresponding components and the amount of the volatile component can affect the absolute content of olfactory sensitivity components rather than the relative content. In a word, the ratios of the olfactory sensitivity components are relatively stable. Due to the fact metal oxide sensors have selectivity, the ratios of the different response intensities of sensors to some extent reflect the ratios of the olfactory sensitivity components. In this study we considered the maximum response for data processing and the inter-ratios of different sensors were calculated. Then we tried to combine the maximum responses of the sensors with their inter-ratios, which was called feature extraction method 2 (FEM2). Figure 5 shows the Radar plots for samples of the Yunnan group and Gansu group. It shows that the Yunnan group and Gansu group were nearly on the same track in the right part of the Radar plots where the positive reactions are shown, but in the left part where the negative reactions are reflected, the Yunnan group and Gansu group were not on the same track. The left parts indicated that there are many differences between them, so in FEM2, only the negative reactions data were calculated.

#### 3.2.1. Raw Data Analysis

According to producing provinces, samples were divided into two groups, Gansu group and Yunnan group. The average value of the ratio of the maximum response of the sensors and RSD of each group were calculated. The results were shown in Table 7. The maximum of the absolute value of RSD of *Danggui* in the Gansu group was just 15.299%, and there were four RSDs below 5%. The results indicated that the differences in the Gansu group were narrowed.

#### 3.2.2. Discrimination of Samples Using PCA

**Figure 6.**
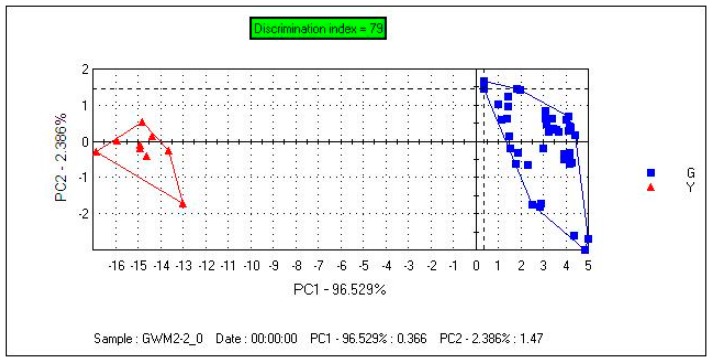
Principal component analysis (PCA) scatter plot for samples (FEM2).

Figure 6 depicts the samples plot. The contribution rates of PC1 and PC2 were 96.529% and 2.386%. The total contribution rate of PC1 and PC2 was 99.915%. A clear separation of samples was observed in the PCA plot. From PC1, we can see that the distribution of samples in the Gansu group was very concentrated. Samples can be clearly separated with PC1, and the distance between the two groups was large compared with Figure 2.

#### 3.2.3. Discrimination of Samples Using SIMCA

**Figure 7.**
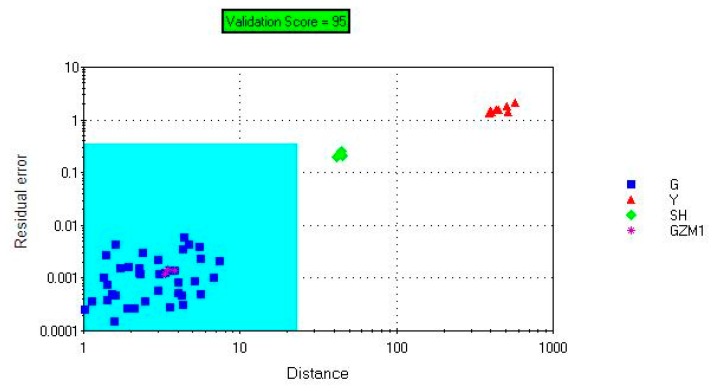
Sample GZM1 and sample SH projected onto the SIMCA model (FEM2).

Figure 7 shows that the model's checking score was 100, which indicated that it was a valid model. The model separated the samples into two regions. The Gansu group was in the acceptable region, and the Yunnan group was outside the acceptable region. GZM1, SH selected as the unknown samples were projected onto the SIMCA model. Figure 6 shows that Sample GZM1 was correctly projected onto the acceptable region and sample SH was correctly projected onto the unacceptable region, which indicated that the model was accurate enough for unknown samples.

#### 3.2.4. Discrimination of Samples Using HCA

**Figure 8.**
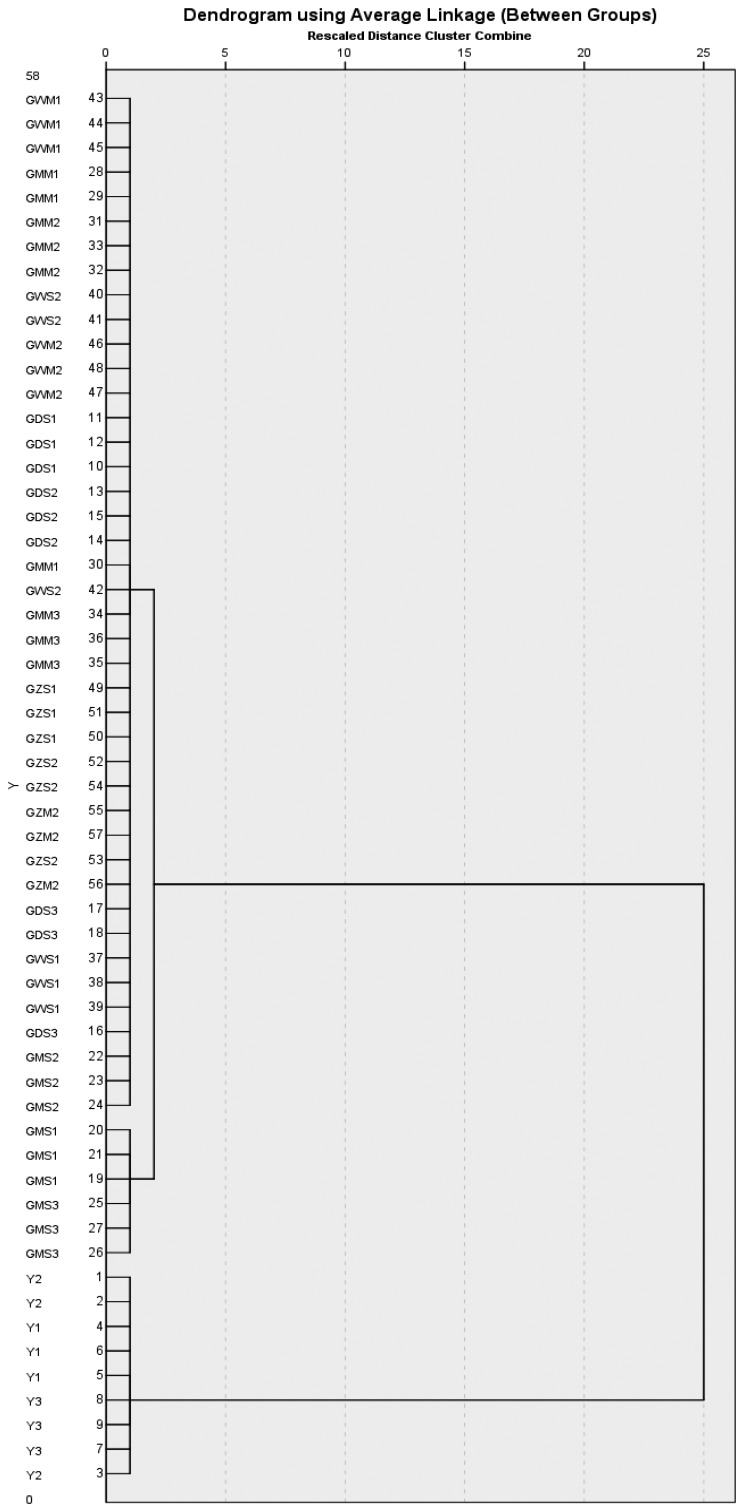
Hierarchical clustering analysis (HCA) dendrogram for samples (FEM2).

Figure 8 depicts the HCA dendrogram. The samples could be divided into two main different groups, the Gansu group and Yunnan group. The Gansu group included GDS, GMS, GMM, GWS, GWM, GZS and GZM, while the other contained Y1, Y2 and Y3. The results showed that the Yunnan group and Gansu group could be clearly separated.

## 4. Conclusions

With an optimized feature extraction method (FEM2), an electronic nose coupled with PCA, SIMCA and HCA is able to objectively analyze and successfully differentiate between Gansu group and Yunnan group *Danggui* samples. In conclusion, the use of an electronic nose in the discrimination between authentic region and unauthentic *Danggui* regions of origin is superior to the traditional methods. It provides a rapid, nondestructive, and accurate method for the categorization of complex aroma mixtures.

## Figures and Tables

**Table 1.** Sample details.

**NOs**	**Place**	**Origin**	**Mark**	**Sample Name**
1	Yunnan Province	Dali	Single root	Y1
2	Yunnan Province	Dali	Single root	Y2
3	Yunnan Province	Lijiang	Single root	Y3
4	Gansu Province	Dangchang	Single root	GDS1
5	Gansu Province	Dangchang	Single root	GDS2
6	Gansu Province	Dangchang	Single root	GDS3
7	Gansu Province	Minxian	Single root	GMS1
8	Gansu Province	Minxian	Many roots	GMM1
9	Gansu Province	Minxian	Single root	GMS2
10	Gansu Province	Minxian	Many roots	GMM2
11	Gansu Province	Minxian	Single root	GMS3
12	Gansu Province	Minxian	Many roots	GMM3
13	Gansu Province	Weiyuan	Single root	GWS1
14	Gansu Province	Weiyuan	Many roots	GWM1
15	Gansu Province	Weiyuan	Single root	GWS2
16	Gansu Province	Weiyuan	Many roots	GWM2
17	Gansu Province	Zhangxian	Single root	GZS1
18	Gansu Province	Zhangxian	Many roots	GZM1
19	Gansu Province	Zhangxian	Single root	GZS2
20	Gansu Province	Zhangxian	Many roots	GZM2
21	Shandong Province	Heze	Single root	SH

**Table 2.** The components and main application of sensors of α-FOX3000 EN.

**No.**	**Name**	**Main Application**
S1	LY2/LG	Oxidizing gas
S2	LY2/G	Ammonia,Carbon monoxide
S3	LY2/AA	Ethanol
S4	LY2/GH	Ammonia/Organic amines
S5	LY2/gCTL	Hydrogen sulfide
S6	LY2/gCT	Propane/Butane
S7	T30/1	Organic solvents
S8	P10/1	Hydrocarbons
S9	P10/2	Methane
S10	P40/1	Fluorine
S11	T70/2	Aromatic compounds
S12	PA/2	Ethanol, Ammonia/Organic amines

**Table 3.** The response intensity of sensors at different temperatures.

**Temperture (°C)**	**LY2/LG**	**LY2/G**	**LY2/AA**	**LY2/GH**	**LY2/gCTL**	**LY2/gCT**	**T30/1**	**P10/1**	**P10/2**	**P40/1**	**T70/2**	**PA/2**
40	0.26	−3.12	−3.4	−2.42	−2.43	−0.68	0.88	0.93	0.76	0.91	0.92	0.99
60	0.02	−0.49	−0.53	−0.41	−0.42	−0.09	0.44	0.55	0.36	0.47	0.43	0.68
80	0.02	−0.61	−0.62	−0.53	−0.55	−0.11	0.45	0.59	0.37	0.49	0.47	0.75

**Table 4.** The response intensity of sensors for different times.

**Time (s)**	**LY2/LG**	**LY2/G**	**LY2/AA**	**LY2/GH**	**LY2/gCTL**	**LY2/gCT**	**T30/1**	**P10/1**	**P10/2**	**P40/1**	**T70/2**	**PA/2**
1200	0.09	−1.48	−1.53	−1.21	−1.21	−0.28	0.62	0.7	0.5	0.62	0.62	0.83
900	0.05	−0.82	−0.84	−0.66	−0.66	−0.15	0.76	0.83	0.61	0.78	0.8	0.95
600	0.03	−0.34	−0.36	−0.27	−0.26	−0.06	0.46	0.52	0.37	0.47	0.41	0.58

**Table 5.** Repeatability for sample GZM1.

**Sample**	**LY2/LG**		**LY2/G**		**LY2/AA**		**LY2/GH**		**LY2/gCTL**		**LY2/gCT**	
	**Mean**	**RSD**	**Mean**	**RSD**	**Mean**	**RSD**	**Mean**	**RSD**	**Mean**	**RSD**	**Mean**	**RSD**
GZM-1	0.224	2.187	−1.630	−0.460	−1.820	−0.412	−0.624	−0.785	−0.864	−0.567	−0.130	0.000
	**T30/1**		**P10/1**		**P10/2**		**P40/1**		**T70/2**		**PA/2**	
**Sample**	**Mean**	**RSD**	**Mean**	**RSD**	**Mean**	**RSD**	**Mean**	**RSD**	**Mean**	**RSD**	**Mean**	**RSD**
GZM-1	0.690	0.000	0.500	0.000	0.404	1.213	0.360	0.000	0.698	0.573	0.766	1.044

Note: RSD (%)

**Table 6.** The average value of the maximum responses of the sensors.

**Sample**	**LY2/LG (s1)**		**LY2/G (s2)**		**LY2/AA (s3)**		**LY2/GH (s4)**		**LY2/gCTL (s5)**		**LY2/gCT (s6)**	
	**Mean**	**RSD (%)**	**Mean**	**RSD (%)**	**Mean**	**RSD (%)**	**Mean**	**RSD (%)**	**Mean**	**RSD (%)**	**Mean**	**RSD (%)**
Gansu group	0.190	12.815	−1.200	− 22.489	− 1.320	− **23.583**	−0.482	−16.996	−0.652	−18.448	−0.094	−24.271
Yunnan group	0.379	10.293	−0.622	−8.697	−0.876	n10.231	−0.958	−14.118	−1.030	−11.655	−0.054	−12.580
**Sample**	**T30/1 (s7)**		**P10/1 (s8)**		**P10/2 (s9)**		**P40/1 (s10)**		**T70/2 (s11)**		**PA/2 (s12)**	
	**Mean**	**RSD (%)**	**Mean**	**RSD (%)**	**Mean**	**RSD (%)**	**Mean**	**RSD (%)**	**Mean**	**RSD (%)**	**Mean**	**RSD (%)**
Gansu group	0.587	13.364	0.389	18.402	0.329	14.859	0.275	19.783	0.566	16.844	0.675	**9.494**
Yunnan group	0.386	8.303	0.214	9.610	0.257	9.365	0.132	11.705	0.329	9.662	0.462	6.351

**Table 7.** The average value of inter-ratios of the maximum responses of the sensors.

**Sample**	**s2/s3**		**s2/s4**		**s2/s5**		**s2/s6**		**s3/s4**	
	**Mean**	**RSD (%)**	**Mean**	**RSD (%)**	**Mean**	**RSD (%)**	**Mean**	**RSD (%)**	**Mean**	**RSD (%)**
Gansu group	0.910	2.112	2.490	12.037	1.840	8.784	12.910	3.992	2.730	12.012
Yunnan group	0.712	1.647	0.655	5.727	0.604	3.344	11.480	4.064	0.919	4.382
**Sample**	**s3/s5**		**s3/s6**		**s4/s5**		**s4/s6**		**s5/s6**	
	**Mean**	**RSD (%)**	**Mean**	**RSD (%)**	**Mean**	**RSD (%)**	**Mean**	**RSD (%)**	**Mean**	**RSD (%)**
Gansu group	2.010	8.705	14.130	4.571	0.742	3.689	5.280	15.299	7.080	11.777
Yunnan group	0.848	2.031	16.130	3.426	0.923	2.568	17.570	4.987	19.030	3.486
